# Dosimetric characterization of CdZnTe radiation detectors under electron-beam irradiation

**DOI:** 10.1371/journal.pone.0349698

**Published:** 2026-06-30

**Authors:** Chansun Park, Jiwon Seo, Sangsu Kim, Hyojung Kim, Jung-Yeol Yeom, Shinhaeng Cho

**Affiliations:** 1 Global Health Technology Research Center, Korea University, Seoul, Republic of Korea; 2 Department of Health and Safety Convergence Science, Korea University, Seoul, Republic of Korea; 3 Department of Radiation Oncology, Chonnam National University Medical School, Gwangju, Republic of Korea; 4 Department of Bioengineering, Korea University, Seoul, Republic of Korea; 5 School of Bioengineering, Korea University, Seoul, Republic of Korea; Harvard School of Public Health, UNITED STATES OF AMERICA

## Abstract

With the growing complexity of modern radiotherapy, the demand for dosimeters capable of delivering real-time, high-resolution, and reliable measurements has grown significantly. Although cadmium–zinc–tellurite (CZT) detectors have emerged as potential solutions to this growing demand, the lack of comprehensive characterization data for these detectors deters their application in clinical electron-beam dosimetry, especially for high-precision tasks such as small-field and point-dose dosimetry. In this study, we systematically characterized CZT detectors, emphasizing the performance trade-offs associated with detector thickness for therapeutic electron-beam dosimetry. The performance of two CZT detectors (6 × 6 mm²), with thicknesses of 5 and 8 mm, was evaluated using a clinical linear accelerator (LINAC) over an electron energy range of 6–18 MeV. Both detectors demonstrated stable baseline performance, including proportional dose linearity (R² > 0.997), dose-rate independence, and excellent long-term stability (<1.3% variation over two months). Furthermore, a distinct thickness-dependent trade-off was identified: while both configurations exhibited highly comparable dose responses at the 6-MeV baseline, the 8-mm detector exhibited better energy capture efficiency for higher-energy electrons. By contrast, the 5‑mm detector offered enhanced angular isotropy with respect to the gantry angle as well as reduced signal variation (±3.5% vs. ± 6.4%), emerging as more suitable for rotational therapies and geometrically demanding scenarios. Additionally, a nonmonotonic energy dependence was observed, characterized by a pronounced response peak at 15 MeV, which was likely attributable to bremsstrahlung photon contamination from the LINAC head. These findings demonstrate that detector thickness serves as an application-specific optimization factor for sensitivity and angular fidelity, offering a practical framework for deploying CZT detectors in both initial clinical evaluations and challenging extra-clinical radiation environments.

## Introduction

Accurate radiation dosimetry is the foundation of modern science and medicine, ensuring the safety and efficacy of applications ranging from cancer therapy to space exploration [1,2]. This method is applied to verify whether prescribed doses are delivered precisely to target volumes while minimizing exposure to surrounding healthy tissues; therefore, it provides a quantitative basis for ensuring the safety and efficacy of clinical treatments. Over the past decades, various dosimetric technologies have been developed, including ionization chambers, thermoluminescent dosimeters (TLDs), and radiochromic films [3,4]. In clinical practice, the advent of sophisticated techniques, such as intensity-modulated radiation therapy (IMRT) and electron-beam therapy for superficial tumors, has compelled the development of dosimeters that can overcome the limitations of conventional systems. Among such devices, ionization chambers are widely employed as reference standards because of their inherent stability, reproducibility, and well-established calibration protocols that are traceable to primary standards [5].

However, to address the increasing complexity of modern radiation delivery modalities, such as IMRT, volumetric modulated arc therapy, and particle beam therapy, the development of detectors with enhanced spatial resolution, real-time readout capability, and adaptability to dynamic radiation fields is necessary [6,7]. Electron-beam therapy, which is utilized to treat superficial malignancies, presents significant dosimetric challenges [8,9]. Electron beams are characterized by steep dose gradients near the surface and rapid dose fall-off with increasing penetration depth, necessitating precise spatial dose mapping. In these environments, the finite sensitive volume of ionization chambers leads to a “volume averaging effect” that compromises the accuracy of point-dose measurements [10–12].

While alternative active detectors, such as silicon diodes, offer higher spatial resolution, they are susceptible to cumulative radiation damage, which can alter their sensitivity and necessitate frequent recalibration. Passive detectors such as TLDs and films, although sensitive, lack real-time feedback and involve labor-intensive post-processing, which renders these devices suboptimal for adaptive therapy and high-throughput clinical workflows. The ongoing demand for high-performance dosimeters has spurred interest in compound semiconductor detectors, particularly those utilizing high-Z materials [13–15].

Among these, cadmium–zinc–telluride (CdZnTe or CZT) has recently gained considerable attention as a promising semiconductor material. CZT offers several intrinsic advantages; these include a high effective atomic number (Z ≈ 50) and density (resulting in good stopping power); a wide and tunable bandgap (~1.4–2.2 eV), which enables low-leakage-current operation at room temperature without cryogenic cooling; and a high charge carrier mobility–lifetime (μτ) product, which contributes to efficient charge collection [16–20]. Furthermore, the direct-conversion mechanism of CZT allows for the fabrication of single-layer devices (i.e., planar architectures that operate without complex internal multilayer junctions, such as p-n junctions), enabling extreme miniaturization of the active cell size. Owing to these features, CZT has emerged as a superior alternative to other novel dosimeters (such as those based on solar cells [21]) for applications requiring high spatial resolution, including point-dose measurements in small fields. The potential for miniaturization also offers significant advantages in terms of device portability and integration. Consequently, CZT detectors are widely applied in fields such as nuclear spectroscopy, medical imaging, and space-based instrumentation owing to their robustness and radiation hardness [22–24].

Despite these advantages, a comprehensive characterization of CZT for clinical electron-beam dosimetry has not yet been attempted. Therefore, in this study, we performed a comprehensive dosimetric characterizations of two CZT detectors with different thicknesses (5 and 8 mm). The novelty of this study lies in the systematic evaluation specifically tailored for therapeutic electron beams, along with a direct comparison of detector performance as a function of thickness. Our systematic evaluation of their performance under clinically used electron-beam irradiation reveals, for the first time, critical thickness-dependent trade-offs among the key dosimetric parameters. This study establishes a robust foundation for the clinical deployment of CZT-based dosimeters. Moreover, the presented fundamental data on the response of the detectors to high-energy electrons are expected to support their use in both terrestrial and space-based radiation environments in the future.

## Materials and methods

### Detector system and characterization

The CZT detectors used in this study were fabricated from an indium-doped single-crystal ingot, with a diameter of 1 in, grown using the vertical Bridgman method. To analyze the physical radiation response of the detectors as a function of thickness, the ingot was diced into two samples with a face area of 6 × 6 mm^2^ and thicknesses of 5 and 8 mm. To remove the subsurface damage caused by the dicing process and inherent defects, the samples were subjected to chemomechanical polishing with a 2% Br-MeOH solution in the final step and were subsequently rinsed in deionized water [25,26]. This step was critical for reducing surface roughness and associated leakage currents. Next, a uniform gold contact layer, with a thickness of at least 50 nm, was deposited on both the polished CZT surfaces to form electrodes. The prepared samples exhibited high bulk resistivities of 1.9 × 10^10^ and 2.1 × 10^10^ Ω·cm, which confirmed their suitability for low-noise operation (S1 and S2 Figs and S1 Data).

The radiation-induced current generated by the CZT detectors under electron-beam irradiation was measured using an electrometer (DOSE1, IBA, Schwarzenbruck, Germany), which was connected via low-noise triaxial cables to minimize electronic interference. The electrometer was operated in the charge integration mode, setting it to a low range to optimize the signal-to-noise ratio for the expected current levels. The detector output was continuously recorded as a function of delivered dose during irradiation. Charge integration was synchronized with the clinical linear accelerator (LINAC) beam delivery to ensure complete signal capture. The measured current was digitally logged on a personal computer in real time at a sampling interval of 400 ms. The total collected charge, obtained via temporal integration of the current signal during the steady-state irradiation period, was used as the dosimetric quantity for subsequent analyses.

### Irradiation facility and dosimetric protocols

**Fig 1:**
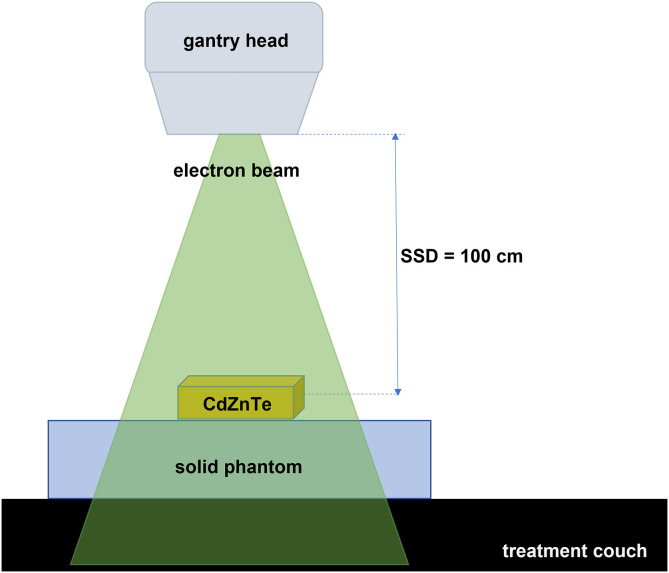
Schematic of the experimental setup for dosimetric measurements. The CZT detector is positioned on a solid phantom and irradiated by an electron beam from a clinical LINAC at an SSD of 100 cm.

All the irradiation experiments were performed using a LINAC (Varian Novalis Tx, Varian Medical Systems, Palo Alto, CA, USA). The CZT detector was positioned on a solid phantom with the source-to-surface distance (SSD) maintained at 100 cm to minimize air gaps. The delivered dose for each irradiation, set in monitor units (MU) (where 1 MU corresponds to 1 cGy under reference conditions), was calibrated to an absolute dose in centigray (cGy) units using a reference ionization chamber (PPC40, IBA Dosimetry, Schwarzenbruck, Germany) and an electrometer (DOSE1, IBA, Schwarzenbruck, Germany) following the IAEA TRS-398 protocol. Measurements were performed at the depth of maximum dose (d_max_) for each electron energy using a field size of 10 × 10 cm^2^. To characterize the energy spectrum of each beam, the reference depth (R_50_) was determined as follows: R_50_ = 2.4, 3.7, 4.9, 6.2, and 7.3 g/cm^2^ for beam energies of 6, 9, 12, 15, and 18 MeV, respectively. Figure 1 illustrates the experimental setup used in this study.

### Dosimetric performance evaluation

Various tests were conducted to evaluate the performance of each detector.

#### Dose linearity and reproducibility.

Dose linearity was evaluated across eight distinct dose points over the range of 10–1000 cGy using a 6-MeV electron beam. The coefficient of determination (R²) was calculated via unweighted linear regression fitting. Short-term reproducibility was quantified using the coefficient of variation (CV) for three consecutive irradiation experiments performed at 200 cGy.

#### Dose-rate dependence.

The dose-rate range of 100–600 cGy/min was selected to align with standard clinical electron-beam delivery protocols at our institution. Notably, the CZT detector demonstrated reliable linear response characteristics in additional measurements conducted up to 1000 cGy·min^−1^. This result confirmed its suitability for high-output LINAC systems. The obtained signal intensities were normalized to the response measured at a dose rate of 400 cGy·min^−1^.

#### Field-size dependence.

The response of the 5-mm-thick CZT detector was measured using a 6-MeV electron beam for square field sizes of 5 × 5, 10 × 10, 15 × 15, 20 × 20, and 25 × 25 cm^2^, each fully covering the CZT detector under investigation. As an initial exploratory evaluation, this investigation was performed only for the 5-mm detector at 6 MeV to isolate fundamental scattering effects while minimizing the influence of complex volume-averaging and high-energy bremsstrahlung contamination, which are more pronounced in thicker detectors or at higher energies. The output for each field size was normalized to the response obtained under a 10 × 10 cm^2^ reference field.

#### Angular dependence.

To assess the isotropic response, the LINAC gantry was rotated from 0° to 315° in 45° steps relative to the central axis of the beam, and the signal variation was measured under constant irradiation of 6 MeV.

#### Energy dependence.

The sensitivity of the detector (signal per unit dose) was measured for each available clinical electron energy (6, 9, 12, 15, and 18 MeV) to characterize its response to different radiation parameters.

### Long-term stability and robustness assessment

To evaluate the long-term reliability and suitability of the detector for applications requiring high radiation tolerance, its stability was monitored over a 2-month period. The dose-linearity measurements (as described in Section 2.3a) were repeated at one-week intervals under identical conditions (6 MeV, 400 cGy·min^−1^), and the consistency of the linear slope (sensitivity) over time was analyzed to quantify the long-term stability of the detector against cumulative radiation exposure.

### Statistical analysis

All the measurements were repeated three times for each detector to ensure stability and precision. While n = 3 provides an estimate of short-term repeatability rather than full statistical variance, a fixed interval of 2 min was maintained between consecutive irradiations to mitigate potential charge-trapping effects. The data points presented in the subsequent figures represent the mean of these three independent measurements, and the error bars indicate the corresponding standard deviations. The linearity of the detector response was quantified by the coefficient of determination (R²) derived from linear regression fitting to the mean values. Short-term reproducibility was assessed by calculating the CV of the three repeated measurements under identical irradiation conditions. All statistical analyses were performed using OriginPro 2016 software (OriginLab Corporation, USA).

## Results

### Fundamental dosimetric characteristics

We selected detector thicknesses of 5 and 8 mm to investigate the dosimetric responses of the CZT detectors in different energy-deposition regimes. The interaction of an electron beam with a detector is fundamentally governed by its continuous slowing-down approximation (CSDA) range, which defines the average path length traveled by an electron prior to stopping. The total stopping-power data for CZT (calculated and obtained from the NIST ESTAR database [27]) were used to determine the CSDA range values via numerical integration [28,29]. The clinical energy ranges used in this study are presented in Table 1.

**Table 1:** Estimated electron-beam range in the CZT detector at different energies.

Beam energy (MeV)	6	9	12	15	18
CSDA range (mm)	5.2	7.9	10.5	13.2	15.9

CSDA, Continuous slowing-down approximation.

The data presented in Table 1 provide critical insights into the underlying physics of the electron beam–detector interaction. For 6-MeV electrons, whose practical range is approximately 5.2 mm, the 5-mm detector captures nearly the entire energy of the incident particle. However, at higher energies (9 MeV and above), the electron range exceeds the thickness of the detector, causing electrons to pass through the crystal while depositing only a fraction of their energy. In contrast, the 8-mm detector is sufficiently thick to completely absorb both 6- and 9-MeV electrons. Electron transit through the detector’s active volume occurs only at beam energies of ≥12 MeV. Therefore, the proposed experimental design is ideal for observing and quantifying the detector response as the interaction mechanism shifts from complete absorption to partial absorption, providing a robust basis for the subsequent comparative analyses. However, while the CSDA range offers a conceptual baseline for energy absorption, the actual dosimetric response in electron-beam therapy is complicated by multiple Coulomb scattering, angular spread, energy straggling, and depth-dependent spectral changes.

### Comparative performance analysis: Effect of detector thickness

Dosimetric evaluations reveal systematic thickness-dependent differences in detector performance. These results are essential for elucidating the interaction of therapeutic electron beams with CZT and for guiding the optimization of detector designs toward targeted applications.

#### Dose linearity.

**Fig 2:**
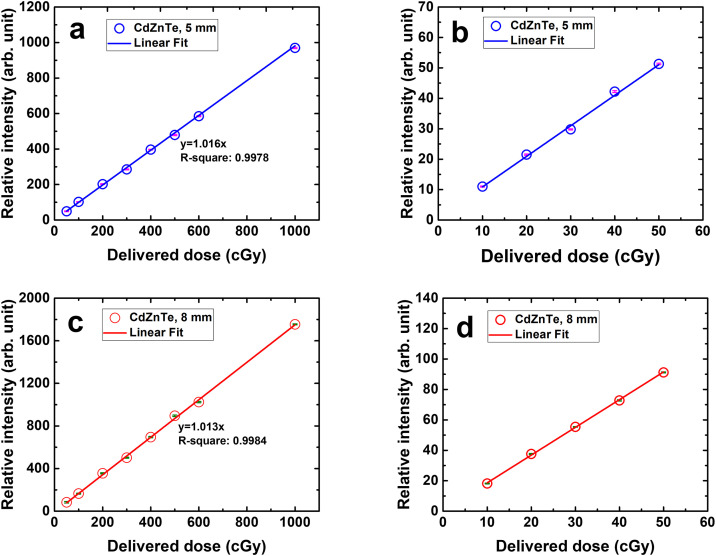
Dose-linearity characterization of 5- and 8-mm CZT detectors using a 6-MeV electron beam. The linear correlation is shown for both the (**a, c**) full therapeutic range and the (**b, d**) expanded low-dose region for the (a, b) 5-mm and (c, d) 8-mm detectors.

Both the 5- and 8-mm-thick CZT detectors demonstrate stable dosimetric performance, exhibiting a highly linear response across the entire therapeutic range of 10–1000 cGy. A linear regression analysis of these detectors yielded R² values of 0.9978 and 0.9984, respectively, confirming their reliable and predictable performance (Fig 2 and Table 2). As summarized in Table 2, the sensitivities derived from the linear regression are highly comparable between the two configurations, yielding 1.016 for the 5-mm detector and 1.013 for the 8-mm detector. This indicates that increasing the thickness from 5 mm to 8 mm does not significantly alter the primary signal sensitivity under 6-MeV irradiation, as both thicknesses match or exceed the CSDA range of the primary electrons. In contrast, prior studies have demonstrated that a thin detector (< 2 mm) cannot maintain a linear response within the therapeutic range of 10–1000 cGy and displays a pronounced sublinear trend beyond this range [17,18]. This deviation from linearity at high doses is attributed to inefficient charge collection caused by the recombination of a high density of charge carriers, which subsequently leads to signal loss. These results demonstrate that thick detectors provide a robust linear response, whereas the operational range of thin detectors under high-flux conditions is limited by saturation effects.

**Table 2:** Summary of dose linearity and fitting parameters for the 5- and 8-mm CZT detectors.

Parameter	5-mm CZT	8-mm CZT
Sensitivity (slope) [nC/cGy]	1.016	1.013
Standard error (slope)	±0.019	±0.016
Intercept [nC]	0.85	1.05
Standard error	±4.22	±3.85
R-square (R^2^)	0.9978	0.9984

CZT, Cadmium–zinc–telluride.

### Dose-rate dependence

**Fig 3:**
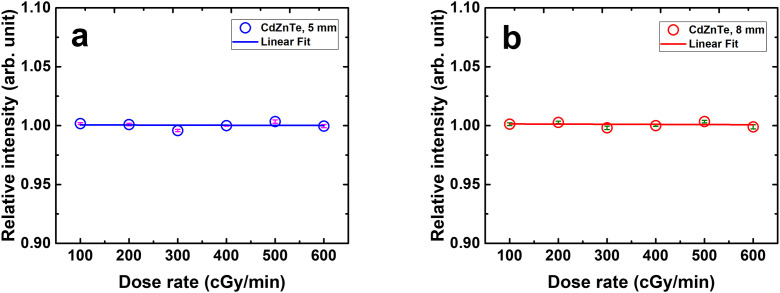
Dose-rate dependence characteristics. Dose-rate dependence of the (**a**) 5-mm and (**b**) 8-mm CZT detectors. The relative signal intensity is plotted as a function of dose rate from 100 to 600 cGy·min^−1^, with all responses normalized to the signal at the 400 cGy·min^−1^ reference rate. Error bars represent the standard deviations of the repeated measurements (n = 3), providing an estimate of short-term repeatability.

Figure 3 shows that the CZT detector provides a stable response within the dose-rate range of 100–600 cGy·min^−1^. The output signal, normalized to the 400 cGy·min^−1^ reference signal, shows no measurable deviation, confirming negligible contributions from charge-carrier recombination or signal pile-up to the detector’s response under these conditions.

#### Sensitivity and field-size dependence.

**Fig 4:**
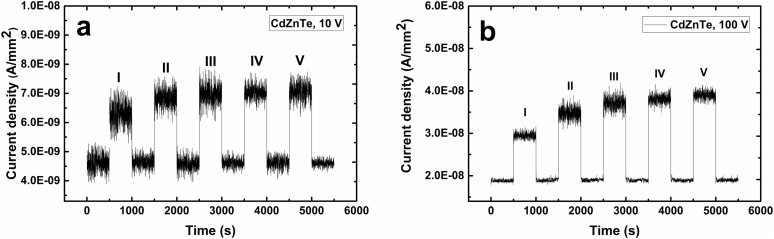
Field-size dependence of the 5-mm CZT detector under 6-MeV electron irradiation at two different voltages. The field-size dependence at **(a)** 10 V and **(b)** 100 V is shown. The relative output factor is plotted for five square field sizes of (I) 5 × 5 cm², (II) 10 × 10 cm², (III) 15 × 15 cm², (IV) 20 × 20 cm², and (V) 25 × 25 cm².

Figure 4 and Table 3 illustrate the dependence of the 5-mm CZT detector’s response on both the field size and applied bias voltage under 6-MeV electron irradiation. The measured current density increases progressively as the field size is increased from 5 × 5 cm² to 25 × 25 cm², primarily owing to the enhanced contributions from phantom- and collimator-scattered radiation. In addition, detector sensitivity is strongly influenced by the applied bias, with higher output signals observed at 100 V compared to those at 10 V. Notably, the magnitude of this field-size dependence is strongly influenced by the applied bias. At 100 V, the relative signal variation is significantly more pronounced than that observed at 10 V, with the output factor ranging from 0.85 to 1.10. This enhancement in signal intensity is attributed to the strong electric field, which improves charge-collection efficiency by reducing carrier trapping and recombination. Consequently, for the same incident radiation dose, detector sensitivity increases with increasing operating voltage.

**Table 3:** Current densities for different field sizes at applied voltages of 10 and 100 V.

Region	I (5 × 5 cm²)	II (10 × 10 cm²)	III (15 × 15 cm²)	IV (20 × 20 cm²)	V (25 × 25 cm²)
Current density (A/mm^2^), 10 V	0.9251	1.0000	1.0235	1.0338	1.0382
Current density (A/mm^2^), 100 V	0.8477	1.0000	1.0661	1.0848	1.0977

All the responses are normalized to the signal measured under the 10 × 10 cm² reference field (II).

#### Angular dependence.

**Fig 5:**
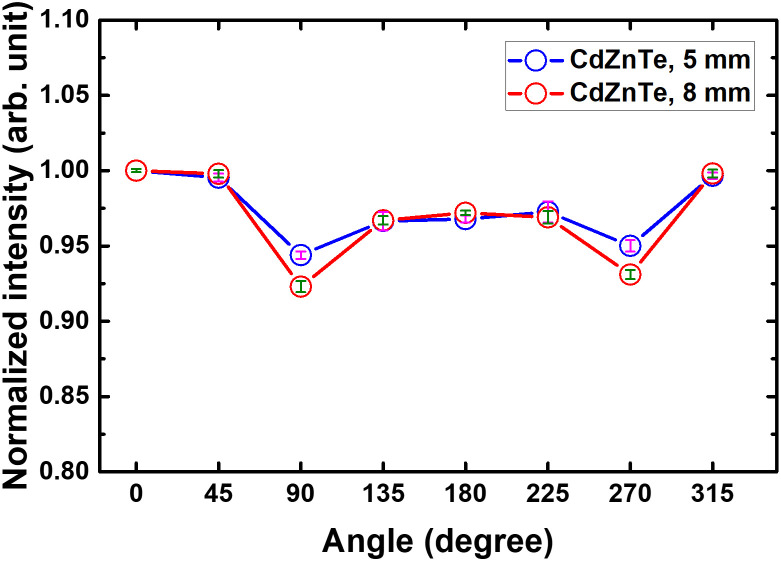
Angular dependence of the detectors. Angular dependence of the (**a**) 5-mm and (**b**) 8-mm CZT detectors under 6-MeV electron irradiation. The relative signal intensity is plotted as a function of gantry angle from 0° to 315°, with all responses normalized to the signal at normal incidence (0°). Error bars represent the standard deviations of the replicated measurements.

The angular dependence of the thin 5-mm CZT detector is smaller than that of the thick 8-mm device (Fig 5). During gantry rotation from 0° to 315° (in 45° steps), the 5-mm detector response varies by less than 3.5%, whereas that of the 8-mm detector shows a maximum deviation of 6.4%. This effect is most pronounced at oblique angles, indicating that angle-correction strategies are required for accurate dosimetry in clinical practice. These results highlight a key trade-off: while increasing the detector thickness improves sensitivity, it also introduces increased angular dependence, thereby affecting reproducibility.

#### Energy dependence.

**Fig 6:**
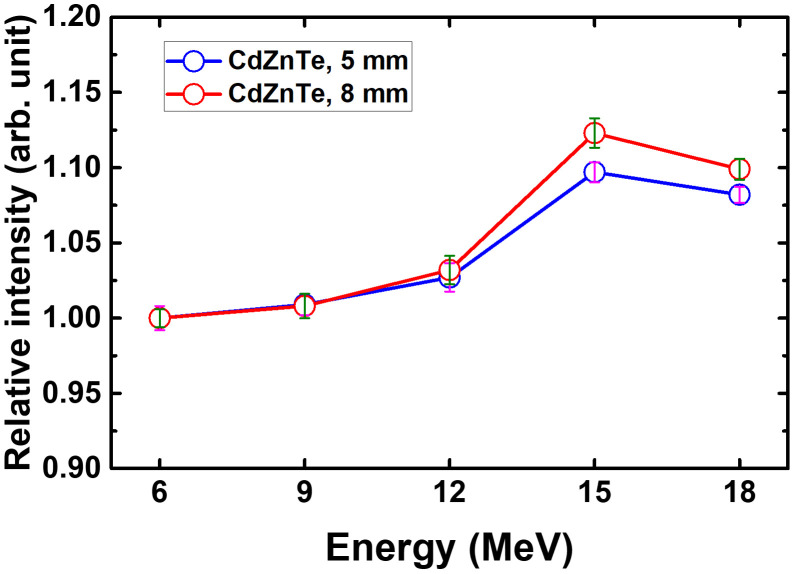
Energy dependence of the 5- and 8-mm CZT detectors. The relative sensitivity (signal per unit dose) is plotted as a function of the nominal electron-beam energy from 6 MeV to 18 MeV. All responses are normalized to the sensitivity measured at 6 MeV. Error bars represent the standard deviations of the replicated measurements.

The energy dependence results, presented in Fig 6 and Table 4, reveal a complex and nonmonotonic trend for both the detectors analyzed in this study. The signal intensities of both the 5- and 8-mm detectors increase with increasing electron-beam energy from 6 MeV to 15 MeV; at 15 MeV, a pronounced and sharp sensitivity peak is detected. The peak observed under 15-MeV beam irradiation is significantly more pronounced for the 8-mm detector than for the 5-mm detector. For the 5- and 8-mm detectors, the signal intensities at 15 MeV are 9.7% and 12.3% higher than those at 6 MeV, respectively. However, with a further increase in beam energy to 18 MeV, the signal intensities of both detectors decrease. These distinct nonlinear responses are attributed to the complex interplay between the energy deposited by the primary electrons and the increasing contribution of secondary bremsstrahlung photons at high energies.

**Table 4:** Comparison of the relative signal intensities shown by the 5- and 8-mm CZT detectors under irradiation by 6-, 9-, 12-, 15-, and 18-MeV electron beams.

Beam energy (MeV)	6	9	12	15	18
CZT, 5 mm	1.000	1.009	1.027	1.097	1.082
CZT, 8 mm	1.000	1.008	1.032	1.123	1.099

CZT, Cadmium–zinc–telluride.

#### Long-term stability and robustness analyses.

**Fig 7:**
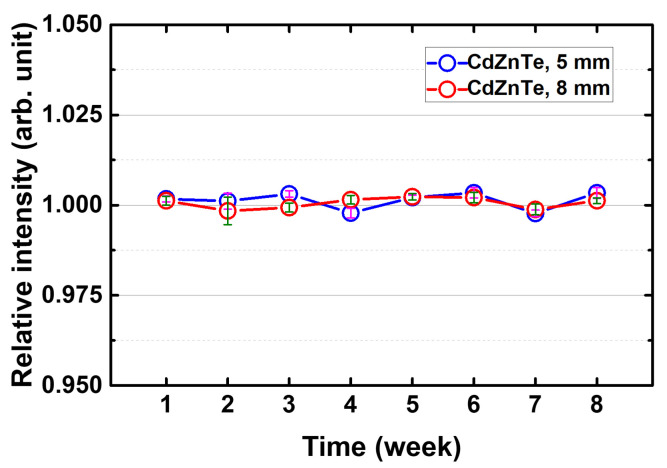
Long-term stability analysis. Long-term stability of the (**a**) 5-mm and (**b**) 8-mm CZT detectors over a 2-month period. The relative sensitivity is plotted as a function of time. All subsequent measured values are normalized to the initial value recorded in the first week to assess performance consistency under cumulative radiation exposure.

Both detectors demonstrated excellent long-term stability throughout the two-month evaluation period, accumulating an estimated total radiation dose of approximately 250 Gy (Fig 7). Sensitivity, defined as the slope of the dose–response linearity curve, was monitored weekly, and the variations remained within 1.3% for both devices. This high consistency highlights the robust radiation hardness of CZT under cumulative electron-beam exposure, emphasizing its suitability for prolonged and repeated operation in demanding clinical environments without frequent recalibration.

## Discussion

The results obtained in this study establish CZT as a highly robust and versatile material for therapeutic electron-beam dosimetry. The stable intrinsic performance of CZT detectors, as demonstrated by their proportional dose linearity (R² > 0.997), dose-rate-invariant response characteristics, and excellent long-term stability (<1.3% variation), position them as reliable devices capable of meeting the demands for accuracy and consistency in high-precision medical applications.

In clinical dosimetry, a critical consideration for CZT is its relatively high effective atomic number (Z ≈ 50), which differs significantly from that of human tissue (Z ≈ 7.4). While many clinical dosimeters are designed to be tissue-equivalent, the non-tissue-equivalent nature of CZT results in energy-dependent detector responses. Nevertheless, the high atomic number of CZT offers improved detection efficiency, enabling more compact detector designs with high spatial resolution.

An important outcome of this study is the elucidation of the performance trade-off directly associated with detector thickness. For the baseline 6-MeV electrons, increasing detector thickness from 5 mm to 8 mm did not yield a significant increase in baseline sensitivity, as a thickness of 5 mm is sufficient to absorb the primary electron energy (CSDA range: ~ 5.2 mm). However, the 8-mm detector exhibits superior energy-capture efficiency for higher-energy electron beams (e.g., > 12 MeV), effectively mitigating the signal loss caused by incomplete electron stopping. Conversely, the 5-mm detector outperforms its thicker counterpart in terms of angular stability, exhibiting substantially lower variations (±3.5% compared with ±6.4% for the 8-mm device). This superior isotropy is advantageous for applications requiring high geometric accuracy, such as the commissioning of rotational therapies and dosimetry in small high-gradient fields. These results provide a clear basis for detector selection: thicker detectors are preferable when maximizing energy capture for high-energy beams is the priority, whereas thinner detectors are more suitable when spatial and angular fidelity are of primary importance.

Furthermore, our results underscore the significance of the operational parameters of CZT detectors. Increasing the applied bias voltage from 10 V to 100 V yields a substantially stronger output signal, which is ascribed to the enhanced charge-collection efficiency provided by the strong internal electric field. This observation indicates that voltage optimization is essential to maximize detector sensitivity as well as to ensure reproducible and reliable detector performance under demanding clinical conditions.

However, the most novel and insightful observation of this study is the anomalous energy dependence, characterized by a sharp response peak at an irradiation dose of 15 MeV, followed by a decrease when the beam energy is increased to 18 MeV. This distinctive feature cannot be explained solely by the electron-stopping power; rather, it is hypothesized to result from the increasing influence of bremsstrahlung contamination generated in the LINAC head [30]. Below 12 MeV, the response is predominantly governed by primary electron interactions. In contrast, at 15 MeV, the bremsstrahlung photon flux is likely to exceed a critical threshold, especially in the 8‑mm detector, resulting in a pronounced signal owing to its large volume, which effectively captures more penetrating photons than does the 5-mm device. The subsequent decline at 18 MeV can be attributed to reduced photon interaction cross-sections, along with increased leakage of high-energy charged particles.

Collectively, these comprehensive characterizations have strong clinical and practical implications. CZT detectors are sensitive to the primary electron dose as well as the mixed radiation field that is intrinsic to LINAC-based treatments. These results indicate that these detectors are suitable for radiotherapy and underscore the need for energy-specific calibration factors to achieve accurate dosimetry.

However, the present study serves as an initial evaluation under controlled laboratory conditions. To advance these findings toward full clinical implementation, further rigorous investigations are necessary. For context, compared with conventional low-Z dosimeters [4,14], thick CZT detectors exhibit distinct physical responses that require careful interpretation.

This study provides a solid foundation that supports the application of CZT in electron-beam dosimetry while highlighting areas that require further refinement before the technology can be broadly implemented in clinical and commercial settings. One such aspect is the angular dependence, which remains a critical limitation and may be mitigated by developing geometric correction algorithms or by improving detector housing and encapsulation to achieve a more isotropic response. In addition, the nontrivial energy dependence, though consistent with established physical principles, poses challenges for dosimetry across a wide energy spectrum. These drawbacks can be mitigated by employing hybrid device-design approaches, such as integrating CZT with complementary materials, to achieve a more energy-independent or tissue-equivalent response. Ultimately, the findings presented in this paper are expected to support such advancements, offering essential baseline data to guide the evolution of CZT from a promising detector material to a platform for the next generation of high-performance dosimeters in both clinical and nonclinical applications.

## Conclusion

Selection of detector thickness in CZT-based electron dosimetry involves a critical, application-specific trade-off between sensitivity and geometric fidelity. In this study, we systematically characterized CZT detectors with two thicknesses (5 and 8 mm). Confirming that while both detectors offered excellent and comparable sensitivities at baseline energies, the thinner detector demonstrated superior angular stability, making it more suitable for complex therapies. Both the detectors demonstrated proportional linearity and stability, supporting their potential for clinical applications. Critically, their energy-dependent responses highlighted their sensitivity to the full mixed radiation field, including contaminant photons, providing essential data for accurate calibration. Ultimately, this study provides a clear framework for tailoring the design of CZT detectors to meet the requirements of initial evaluations in high-precision radiotherapy and other challenging radiation environments, such as space.

## Supporting information

S1 Fig I-V characteristics of a 5-mm-thick CdZnTe detector with dimensions of 6 × 6 × 5 mm^3^. (TIF)

S2 Fig I-V characteristics of an 8-mm-thick CdZnTe detector with dimensions of 6 × 6 × 8 mm^3^. (TIF)

S1 Data Underlying numerical data for the I-V characteristics and resistivity measurements. This file contains the raw data used to characterize the electrical properties of the 5-mm and 8-mm CZT detectors. (CSV)
