# Supplementary figures and images for: Dosimetric characterization of CdZnTe radiation detectors under electron-beam irradiation
Source: PLoS One. 2026 Jun 30;21(6):e0349698. doi: 10.1371/journal.pone.0349698 (PMC13318049; doi:10.1371/journal.pone.0349698)

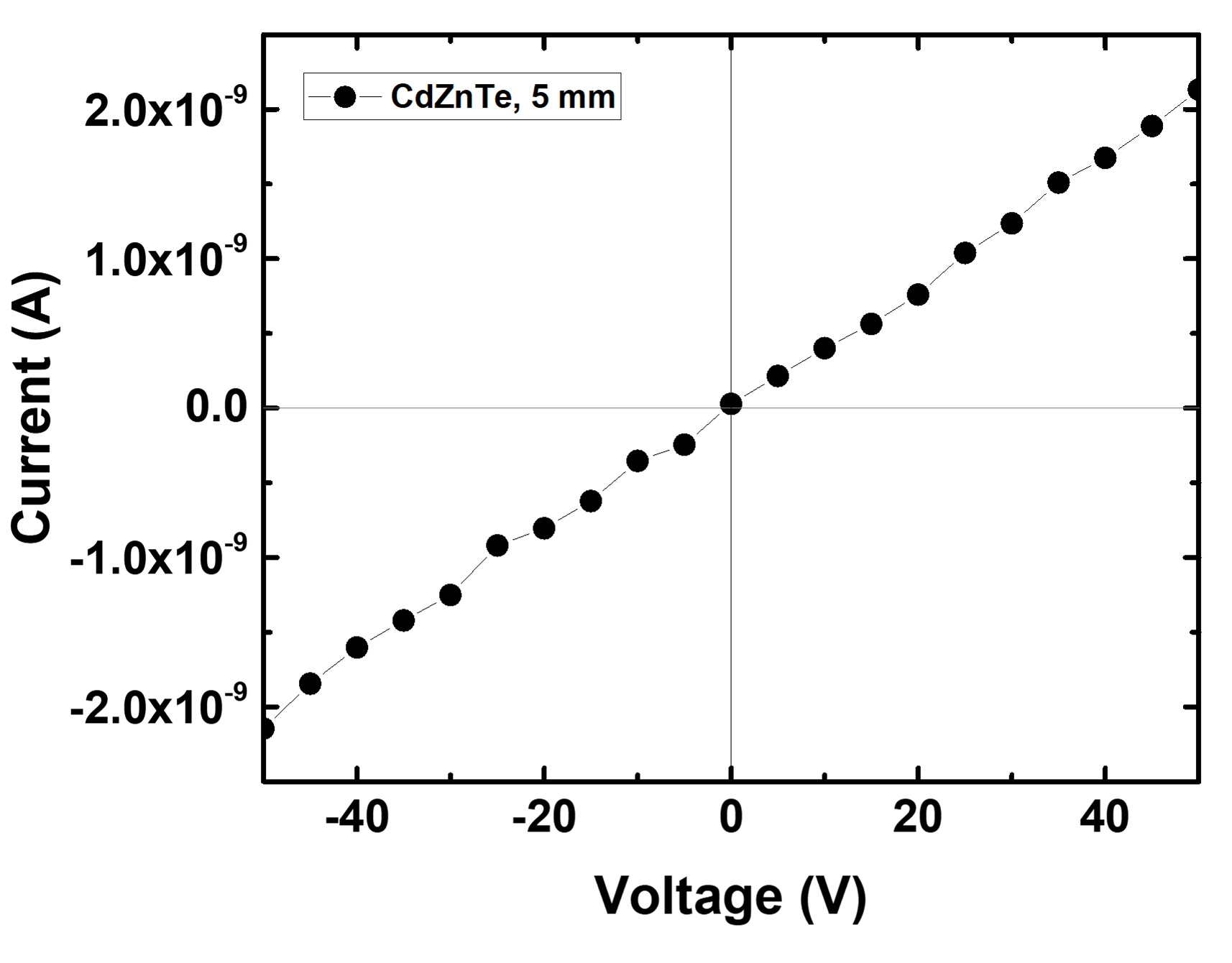

Supplement: S1 Fig — (TIF) [file pone.0349698.s001.tif]

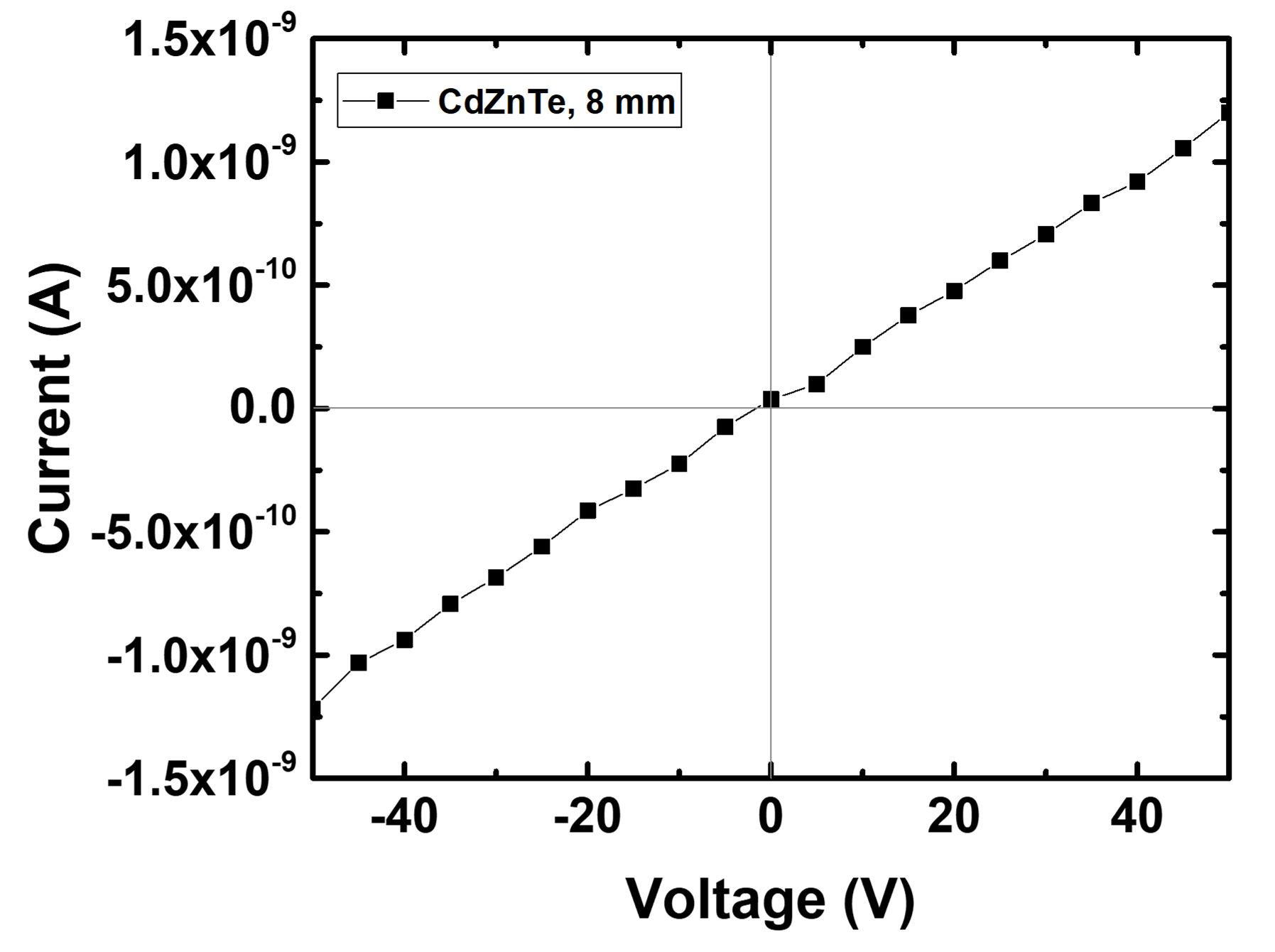

Supplement: S2 Fig — (TIF) [file pone.0349698.s002.tif]
